# Are the Major “Western” Findings on Attitudes to Immigration Supported by Global Evidence? Mostly Yes, Actually

**DOI:** 10.1093/poq/nfag028

**Published:** 2026-07-11

**Authors:** James Dennison

**Affiliations:** Pierre Keller Professor, Harvard Kennedy School, Harvard University, Cambridge, MA, USA; Part-time Professor, Migration Policy Centre, European University Institute, Florence, Italy

## Abstract

The causes of variation in attitudes to immigration have been the subject of extensive scholarship in recent decades, with some factors being repeatedly evidenced. However, these findings are overwhelmingly based on studies from a small selection of “Western” countries. This article makes use of recent World Values Survey data across 66 countries globally to test how generalizable such findings on the determinants of attitudes to immigration are globally. It finds that the most typically identified sociodemographic, economic, and contextual determinants of immigration policy preferences, prejudices, and perceived effects are largely generalizable globally, namely: age, low income, job worries, rurality, not having an immigrant background, social distrust, national migration rate, feeling unsafe in one’s neighborhood, and national media bias. The effects of gender, education, and left-right self-placement are weaker and less generalizable. These findings are robust across world regions. Although the predictive power of all factors tend to be smaller outside of Western Europe, these findings shed light on the more profound, universal, and genuine nature of attitudes to immigration, their likely internal determinants—rather than purely external and contextual ones—and the global reliability of measuring their predictors.

To what extent do the major findings on attitudes to immigration drawn from “Western” countries hold when tested globally? While this may seem like a straightforward question of replicability, it carries three important implications: first, migration’s substantive political and societal relevance far beyond the West; second, the scientific value of genuinely generalizable knowledge on immigration attitudes; and third, the growing necessity—and feasibility—of comparative research now that global survey data are widely available. Explaining variation in attitudes to immigration beyond a handful of “Western” countries can offer deeper insights into key social scientific questions. Yet, the vast majority of studies consider “Western advanced democracies” despite immigration being an important political issue in every region of the world.

Exploring whether major “Western” findings on attitudes to immigration hold globally allows us to test not just a substantively vital question but also the broader challenge of genuinely comparative public opinion research. While the field aspires to generalizability, patterns of public attitudes are inevitably shaped by specific environmental, historical, and political contexts (Esser and Vliegenthart 2017; Rojas and Valenzuela 2019), yet most studies overwhelmingly focus on Western democracies, with influential reviews limiting their scope to North America and Western Europe (Hainmueller and Hopkins 2014, 2015) or interpreting immigration through a specifically Western racial prism (Berg 2015), leading scholars to lament the geographic skew of the literature (Dennison and Vrânceanu 2022). Nevertheless, Western studies consistently identify a set of sociodemographic, economic, attitudinal, and contextual predictors as key determinants of immigration attitudes (Ceobanu and Escandell 2010; Rustenbach 2010; Hainmueller and Hopkins 2014; Hellwig and Sinno 2017; De Coninck 2020; Esses 2021; Abdelaaty and Steele 2022), even as publication incentives and narrow causal approaches have precluded deeper, comparative explanations of attitudinal formation (Dennison and Geddes 2026). This research note takes up the challenge by examining whether these recurrent “Western” insights apply beyond the West using global evidence.

This study is not interested in either exactly specifying the most common findings or the state of the literature regarding those put forward, but rather is instead interested in testing whether, as a general rule, those factors repeatedly shown to have effects in “Western” countries have similar effects elsewhere in the world. Yet to do so requires at least briefly overviewing key findings on each of those, roughly identified, as key factors and split into sociodemographic and economic indicators, on the one hand, and attitudinal and contextual indicators, on the other.

Levels of education have been repeatedly shown to be positively associated with attitudes to immigration, either owing to socialization, various immigration-friendly norms and traits, or greater cognitive ability (Mayda 2006; Hainmueller and Hiscox 2007; d’Hombres and Nunziata 2016; Cavaille and Marshall 2019; Kratz 2021; Margaryan et al. 2021; Velásquez and Eger 2022), simply self-selection (Lancee and Sarrasin 2015), or generational differences (Finseraas et al. 2018; McLaren et al. 2021; Lindskog and Oskarson 2023). Thomsen and Olsen (2017) show that the effect of education on attitudes to immigration is contingent on the country’s democratic history and values.

The effects of youth, female gender, and having an immigrant background are typically considered either as control variables when testing the effects of other theorized factors or as contingencies (Espenshade and Calhoun 1993; Hood et al. 1997; Haubert and Fussell 2006; Branton 2007; Just and Anderson 2015; Helbling and Traunmüller 2016; Huber and Oberdabernig 2016; Ponce 2017). Despite being assumed to have positive effects on attitudes to immigration, they have relatively rarely been tested as the main factor of interest. More recently, studies have shown that there is little gender difference, though women were found to hold more anti-Muslim views in Europe (Ponce 2017). Worse or worsening economic positions are similarly often assumed—and shown—to have negative effects on attitudes to immigration. These can be via objective indicators like income (Becchetti et al. 2010; Naumann et al. 2018; Abdelaaty and Steele 2022) or subjective indicators regarding worries and fears (Pardos-Prado and Xena 2019; Polavieja 2016; Burns and Gimpel 2000; Ackermann and Freitag 2015; Melcher 2021).

On attitudinal and contextual indicators, social trust—the extent to which one believes that people generally, including strangers, can be trusted—has been repeatedly shown to have a positive effect on attitudes to immigration in Europe (Herreros and Criado 2009; Rustenbach 2010; van der Linden et al. 2017; Sipinen et al. 2020; Fierro and Parella 2023). Various measures of nationalism, national identity, narcissism, and closeness to one’s country have been shown to be negatively associated with attitudes to immigration (Lindstam et al. 2021; Taniguchi 2021; Górska et al. 2022; Verkuyten et al. 2022). Immigration attitudes are regularly shown to correlate with ideological outlook, left-right self-placement, and political values such as individualism, egalitarianism, and humanitarianism (Pantoja 2006).

Regarding the much-theorized negative effects of media bias, Štětka et al. (2021; also Brader et al. 2008; Schlueter and Davidov 2013; Héricourt and Spielvogel 2014; Van Klingeren et al. 2015; Eberl et al. 2018) find that being exposed to public service media reduces negativity whereas exposure to more diverse and nonmainstream news sources can increase negativity. Urbanity has also been repeatedly shown to be positively associated with attitudes to immigration, in line with the supposed socialization effects of experiences of immigration-related diversity early in life (McLaren et al. 2021; Schmidt 2021; Eger et al. 2022) and sorting by education (Maxwell 2019). Finally, various studies in Europe and North America have *tended to* show that increasing migration rates are associated with worsening attitudes to immigration, with a contrary association for stocks (Schmidt 2021; Claassen and McLaren 2022; Margalit and Solodoch 2022). Finally, feelings of neighborhood safety and concerns about social disorder and crime have been repeatedly shown to relate to immigration attitudes (Semyonov et al. 2012; Dennison and Talò 2017; Turper 2017; Van Heerden and Ruedin 2019; Di Mauro and Memoli 2021).

Although studies on attitudes to immigration have overwhelmingly used “Western” data, they have not exclusively done so (e.g., Mayda 2006; Van Klingeren et al. 2015; Haynes et al. 2016; Tomiura et al. 2019; Dennison and Geddes 2021; Taniguchi 2021; Joshanloo 2024), with those from elsewhere at times providing findings contrary to the most commonly found effects as outlined above. Evidence from early in the twenty-first century in Europe suggested that women tended to oppose immigration more than men (O’Rourke and Sinnott 2006), though this was primarily due to results in Central and Eastern European countries. Mayda (2006; also Barceló 2016) shows that the positive effect of relative income, using global data from the World Values Study, is not statistically significant, while Miller (2012) uses global data to show that self-assessed poverty, unemployment, and, surprisingly, income all have negative effects on attitudes to immigration. Gordon and Maharaj (2015) show that in South Africa social bonds are more effective predictors of attitudes to immigration than social trust.

Generalizability is defined here as a shared direction and broadly comparable effect size across Western and non-Western models, rather than reliance on statistical significance alone.

## Methods and Data

Systematically comparing the determinants of attitudes to immigration in “Western” and non-Western countries requires using global data that includes both relevant measures of such attitudes and measures of the factors outlined above. For this, the World Values Survey (WVS) is the ideal data source (Haerpfer et al. 2020). The WVS’s most recently fully completed wave—its seventh—comprises 66 countries^1^ and societies conducted between 2017 and 2022 and more than 80,000 respondents. The WVS is conducted globally every five years and is noted for its representative, comparative social survey questions, using a common questionnaire, and extensive geographical and thematic scope, free availability of survey data, and project findings.

Wave 7 of the WVS includes multiple questions on attitudes to immigration—including policy preferences, prejudices, and perceived effects—and the determinants outlined above. Summary statistics of those questions are outlined in table 1. Regarding policy preferences, it asks, “How about people from other countries coming here to work. Which one of the following do you think the government should do? 1. Let anyone come who wants to; 2. Let people come as long as there are jobs available; 3. Place strict limits on the number of foreigners who can come here; 4. Prohibit people coming here from other countries.” Regarding prejudice, it asks, “On this list are various groups of people. Could you please mention any that you would not like to have as neighbors? Immigrants/foreign workers.” Regarding perceived effects, it asks, “Now we would like to know your opinion about the people from other countries who come to live in [your country]—the immigrants. How would you evaluate the impact of these people on the development of [your country]? 5. Very good; 4. Quite good; 3. Neither good nor bad; 2. Quite bad; 1. Very bad.” Additionally, though not analyzed in this note, the WVS asks about agreement regarding eight narratives regarding immigration, four good and four bad (see Dennison and Geddes 2026), and agreement whether “When jobs are scarce, employers should give priority to people of this country over immigrants.” Details on the explanatory variables can be found on the WVS website and are mostly self-explanatory; media bias is included in the WVS, though originally taken from the V-Dem 2019 data.

**Table 1.** Summary statistics of dependent and independent variables.

Variable	Obs.	Mean	SD	Min	Max
Attitudes to immigration					
Q130. Restrictive immigration policy preference	91,712	2.56	0.81	1	4
Q21. Anti-immigrant prejudice	95,914	0.22	0.41	0	1
Q121. Belief immigration been good for country’s development	94,687	2.98	1.06	1	5
Sociodemographic and economic predictors					
(coded by WVS) Rurality	97,183	1.32	0.47	1	2
Q275R. Education	96,149	2.20	0.81	1	3
Q288. Income decile	94,259	4.91	2.09	1	10
Q142. Job worries	92,777	2.20	1.11	1	4
Q260. Gender (female)	97,125	0.53	0.50	0	1
Q263. Immigrant	96,836	0.06	0.23	0	1
Q264/Q265 At least one immigrant parent	92,392	0.12	0.32	0	1
Q262. Age	96,709	43.18	16.58	16	103
Attitudinal and contextual predictors					
Q57. Social trust	95,883	0.24	0.43	0	1
Q257. Feel close to country	96,041	3.27	0.80	1	4
Q240. Left-right self-placement	69,436	5.67	2.43	1	10
(coded by WVS) National migration rate	92,396	0.81	4.49	−22.3	22.8
(V-Dem 2019) Media bias	93,619	0.72	1.18	−2.87	2.94
Q131. Feel safe in neighborhood	96,633	3.45	0.87	1	4

With this data, I present several tables reporting the results of regression analyses predicting policy preferences, prejudice, and perceived effect on development. To assess whether the three outcome variables—policy preferences, prejudice, and perceived development impact—reflect a single underlying attitude, I ran a principal factor analysis (PFA) on the pooled sample (*N* ≈ 89,012) and inspected pairwise correlations. The PFA’s likelihood ratio test confirmed the items are correlated (χ^2^(3) ≈ 10,558, *p* < 0.001), but the low first eigenvalue (0.47), modest and inconsistent factor loadings (0.42, 0.31, and –0.45), and high uniqueness scores (≥ 0.80) indicate that a single common factor explains little of their variance. Pairwise correlations are modest—r = 0.13 (policy–prejudice), –0.28 (policy–development), and –0.17 (prejudice–development), all *p* < 0.001—underscoring related but distinct constructs. These results do not support collapsing the outcomes into one dimension, justifying their separate modeling, in line with theoretical expectations.

In each table, Western Europe and English-speaking countries—Andorra, Australia, Canada, Germany, Great Britain, Netherlands, New Zealand, Northern Ireland, and the United States—are analyzed separately and alongside all other countries in the WVS, including those in Central and Eastern Europe, the Balkans, and the former Soviet Union. Given the, presumably, more distal nature of sociodemographic and, to a lesser extent, economic predictors, these are used to predict each of the three types of attitudes to immigration alone and then alongside the psychological and contextual variables, as outlined in table 1. The former includes “Feeling close to one’s country,” which acts as a partial proxy for strength of national identity, being a recognized and tested measure of patriotism and closely associated with nationalism (Ariely 2020). In Appendix A, I offer further regressions that use different weighting schemes and divide the sample countries into nine world regions. All models include dummy variables for each country.

## Results

### Policy Preferences

Table 2 reports results from regression analyses predicting restrictive immigration policy preferences in Western Europe and the English-speaking world (columns 1 and 3) and the rest of the world (columns 2 and 4). Models 1 and 2 include only sociodemographic and economic predictors. Across both regions, tertiary education, higher income decile, lower job worries, immigrant background (first and second generation), and older age are all associated with less restrictive preferences, with *p*-values below 0.05 in each case. The only variable with opposite signed effects across regions is gender: Being female predicts less restrictive preferences in Western countries but more restrictive preferences elsewhere.

**Table 2.** Four linear regression models predicting restrictive immigration policy preferences.

	(1)	(2)	(3)	(4)
	Restrictive preferences			
	Sociodemographics and economics		Add psychology and context	
	W. Europe and English-speaking	Rest of world	W. Europe and English-speaking	Rest of world
Education (ref: none/primary)				
Secondary	−0.05	−0.01	−0.04	0.00
	(0.02) [0.06]	(0.02) [0.59]	(0.03) [0.24]	(0.02) [0.98]
Tertiary	−0.24	−0.05	−0.16	−0.04
	(0.04) [0.00]	(0.03) [0.07]	(0.03) [0.00]	(0.03) [0.23]
Income decile	−0.01	−0.02	−0.01	−0.01
	(0.01) [0.28]	(0.00) [0.00]	(0.01) [0.33]	(0.00) [0.00]
Job worries	−0.05	−0.03	−0.04	−0.03
	(0.01) [0.00]	(0.01) [0.00]	(0.01) [0.00]	(0.01) [0.00]
Female	−0.03	0.02	−0.01	0.02
	(0.01) [0.05]	(0.01) [0.03]	(0.02) [0.44]	(0.01) [0.03]
Immigrant	−0.06	−0.12	−0.07	−0.13
	(0.02) [0.03]	(0.03) [0.00]	(0.01) [0.00]	(0.03) [0.00]
Immigrant parent	−0.11	−0.05	−0.10	−0.04
	(0.02) [0.00]	(0.03) [0.10]	(0.03) [0.01]	(0.04) [0.30]
Age	0.01	0.00	0.01	0.00
	(0.00) [0.00]	(0.00) [0.00]	(0.00) [0.00]	(0.00) [0.00]
Social distrust			0.21	0.17
			(0.02) [0.00]	(0.03) [0.00]
Feel close to country			−0.01	−0.01
			(0.03) [0.73]	(0.01) [0.36]
Left-right self-placement			0.07	0.00
			(0.01) [0.00]	(0.00) [0.74]
National migration rate			0.01	0.02
			(0.00) [0.12]	(0.00) [0.00]
Media unbias			−0.18	−0.22
			(0.01) [0.00]	(0.01) [0.00]
Feel safe in neighborhood			−0.03	0.03
			(0.02) [0.09]	(0.01) [0.01]
Rurality			0.03	0.04
			(0.03) [0.25]	(0.02) [0.04]
Country dummy variables	YES	YES	YES	YES
SE clustered by country	YES	YES	YES	YES
Individual weight	YES	YES	YES	YES
Population weight	NO	NO	NO	NO
Constant	2.38	2.42	1.94	2.22
	(0.04) [0.00]	(0.03) [0.00]	(0.15) [0.00]	(0.06) [0.00]
Observations	13,030	68,867	11,662	45,177
*R*-squared	0.08	0.09	0.15	0.09

*Note:* Coefficients with standard errors in parentheses and exact *p*-values in brackets. Variables unstandardized.

Models 3 and 4 add attitudinal and contextual variables. Social distrust and perceived media unbias are positively associated with restrictive preferences in both samples, while feeling safe in one’s neighborhood is negatively associated in Western countries only. Left-right ideological self-placement and national migration rates are positively signed in both models but fall below the 0.05 threshold in one of the two models. Feeling close to one’s country has *p*-values above 0.05 in both cases. Rurality shows a small, positive association in both samples, with *p*-values just above the cutoff. Overall, most predictors display consistent directional effects across regions, even when *p*-values differ. This points to a broadly shared set of correlates, with some region-specific variation in the strength and clarity of associations.

Table A1 reproduces the four models using cross-national population weights. The coefficients are substantively very similar to those in table 2, and the main conclusions are unchanged, although overall model fit is slightly lower in the population-weighted specifications, particularly outside Western Europe. This becomes clearer in tables A2 and A3, which disaggregate the analysis into nine world regions. In table A2 (sociodemographic and economic predictors only), Western Europe stands out with the highest explained variance (R^2^ = 0.16), whereas the English-speaking bloc has an R^2^ of 0.07—similar to Central/Eastern Europe, the Middle East and North Africa, and South/Southeast Asia. Several non-Western regions (e.g., the former Soviet states and Latin America) display higher explanatory power than the English-speaking group. I use these models to further test for multicollinearity: Across the nine models, no variable has a VIF score above 3.8 and no model has a mean VIF higher than 1.87, well below the typical concern thresholds of 5 or 10.

**Table 3.** Four linear regression models predicting anti-immigrant prejudice.

	(1)	(2)	(3)	(4)
	Anti-immigrant prejudice			
	Sociodemographics and economics		Add psychology and context	
	W. Europe and English-speaking	Rest of world	W. Europe and English-speaking	Rest of world
Education (ref: none/primary)				
Secondary	−0.03	−0.02	−0.02	−0.03
	(0.01) [0.08]	(0.01) [0.00]	(0.01) [0.28]	(0.01) [0.00]
Tertiary	−0.06	−0.04	−0.04	−0.04
	(0.02) [0.00]	(0.01) [0.00]	(0.01) [0.02]	(0.01) [0.00]
Income decile	0.00	−0.00	0.00	−0.00
	(0.00) [0.52]	(0.00) [0.16]	(0.00) [0.15]	(0.00) [0.34]
Job worries	−0.01	−0.00	−0.00	−0.00
	(0.00) [0.01]	(0.00) [0.11]	(0.00) [0.14]	(0.00) [0.41]
Female	−0.03	0.00	−0.02	0.00
	(0.01) [0.01]	(0.00) [0.50]	(0.01) [0.01]	(0.01) [0.90]
Immigrant	−0.03	−0.03	−0.04	−0.03
	(0.01) [0.01]	(0.01) [0.03]	(0.01) [0.01]	(0.02) [0.04]
Immigrant parent	−0.04	−0.03	−0.03	−0.01
	(0.01) [0.00]	(0.01) [0.04]	(0.01) [0.00]	(0.01) [0.56]
Age	0.00	0.00	0.00	0.00
	(0.00) [0.34]	(0.00) [0.00]	(0.00) [0.41]	(0.00) [0.01]
Social distrust			0.05	0.00
			(0.01) [0.00]	(0.01) [0.75]
Feel close to country			0.01	−0.00
			(0.01) [0.07]	(0.01) [0.54]
Left-right self-placement			0.02	0.00
			(0.00) [0.00]	(0.00) [0.00]
National migration rate			0.00	−0.00
			(0.00) [0.00]	(0.00) [0.00]
Media unbias			−0.03	−0.00
			(0.00) [0.00]	(0.00) [0.53]
Feel safe in neighborhood			−0.03	−0.02
			(0.01) [0.01]	(0.01) [0.01]
Rurality			0.01	0.01
			(0.01) [0.45]	(0.01) [0.45]
Country dummy variables	YES	YES	YES	YES
SE clustered by country	YES	YES	YES	YES
Individual weight	YES	YES	YES	YES
Population weight	NO	NO	NO	NO
Constant	0.13	0.04	0.01	0.05
	(0.02) [0.00]	(0.01) [0.00]	(0.02) [0.79]	(0.05) [0.31]
Observations	13,092	69,492	11,657	45,192
*R*-squared	0.05	0.12	0.08	0.11

*Note:* Coefficients with standard errors in parentheses and exact *p*-values in brackets. Variables unstandardized.

### Prejudice

Table 3 presents four regression models predicting anti-immigrant prejudice, following the same two-stage modeling strategy as in table 2. In models 1 and 2, which include only sociodemographic and economic predictors, several variables show consistent associations across “Western Europe and English-speaking” countries and the “Rest of the World.” Secondary and tertiary education, job worries, immigrant status, immigrant parentage, and age all display *p*-values below 0.05 in both models and in the same direction, indicating a similar pattern of association across the two settings. Rurality is signed in the same direction but falls below the threshold only outside the West, while income and gender each cross the threshold in only one model, and in opposite directions. As in table 2, the explanatory power of the models differs noticeably: Model 2 (Rest of World) accounts for more than twice the variance (R^2^ = 0.12) of model 1 (R^2^ = 0.05).

When attitudinal and contextual variables are added in models 3 and 4, the set of shared predictors narrows. Only left-right self-placement and feeling safe in one’s neighborhood have *p*-values below 0.05 in both models and in the same direction. Other attitudinal and contextual variables—such as social distrust, closeness to country, national migration rates, and perceptions of media bias—either fall below the threshold in only one model or change sign, suggesting that their effects are less generalizable across world regions. Even so, the broader pattern holds: A cluster of sociodemographic variables is stable across regions, whereas the relevance of attitudinal and contextual factors varies more by context. Table A4 replicates these models using population weights and produces a broadly similar pattern, with social distrust emerging as the only additional variable to align across both models once weighting is applied.

### Perceptions of Effects of Immigration

Table 4 presents the results of regression models predicting belief that immigration has been good for the country’s development. In models 1 and 2, rurality, education, income, job worries, immigrant status, immigrant parentage, and age are all associated with *p*-values below 0.05 in the same direction across both world regions. Being female has a negative association in both models, but falls below the *p*-value threshold only in the latter. Model 2 again explains more variance than model 1. When attitudinal and contextual predictors are added in models 3 and 4, social distrust, the national migration rate, and media unbias are the only variables that retain associations with *p*-values below 0.05 in the same direction across both regional samples. Other predictors differ in strength or direction across models, including feeling close to one’s country, left-right self-placement, and neighborhood safety. The population-weighted models in table A5 replicate the same pattern of shared predictors, with the addition that feeling close to one’s country also reaches the *p*-value threshold in both models (negatively associated). Rurality remains weak in the extended models in both tables.

**Table 4.** Four linear regression models predicting belief that immigration has been good for the country’s development.

	(1)	(2)	(3)	(4)
	Belief immigration has been good for country’s development			
	Sociodemographics and economics		Add psychology and context	
	W. Europe and English-speaking	Rest of world	W. Europe and English-speaking	Rest of world
Education (ref: none/primary)				
Secondary	0.07	0.00	0.00	−0.01
	(0.05) [0.23]	(0.02) [0.96]	(0.07) [0.95]	(0.02) [0.60]
Tertiary	0.30	0.04	0.17	0.03
	(0.07) [0.00]	(0.02) [0.08]	(0.08) [0.95]	(0.03) [0.22]
Income decile	0.02	0.02	0.02	0.02
	(0.01) [0.06]	(0.00) [0.00]	(0.01) [0.14]	(0.01) [0.00]
Job worries	0.06	0.03	0.04	0.03
	(0.02) [0.01]	(0.01) [0.00]	(0.01) [0.00]	(0.01) [0.01]
Female	−0.02	−0.03	−0.05	−0.03
	(0.03) [0.50]	(0.01) [0.06]	(0.03) [0.14]	(0.01) [0.04]
Immigrant	0.21	0.23	0.22	0.28
	(0.05) [0.00]	(0.06) [0.00]	(0.06) [0.01]	(0.08) [0.00]
Immigrant parent	0.12	0.09	0.11	0.12
	(0.05) [0.05]	(0.03) [0.00]	(0.05) [0.07]	(0.02) [0.00]
Age	−0.00	−0.00	−0.00	−0.00
	(0.00) [0.21]	(0.00) [0.00]	(0.00) [0.15]	(0.00) [0.00]
Social distrust			−0.25	−0.10
			(0.08) [0.02]	(0.03) [0.00]
Feel close to country			−0.09	−0.00
			(0.03) [0.04]	(0.01) [0.97]
Left-right self-placement			−0.09	−0.00
			(0.02) [0.01]	(0.01) [0.58]
National migration rate			−0.14	−0.02
			(0.00) [0.00]	(0.00) [0.00]
Media unbias			0.44	0.16
			(0.03) [0.00]	(0.01) [0.00]
Feel safe in neighborhood			0.02	−0.00
			(0.03) [0.51]	(0.01) [0.78]
Rurality			−0.03	−0.03
			(0.05) [0.58]	(0.02) [0.29]
Country dummy variables	YES	YES	YES	YES
SE clustered by country	YES	YES	YES	YES
Individual weight	YES	YES	YES	YES
Population weight	NO	NO	NO	NO
Constant	3.44	3.02	4.08	3.08
	(0.14) [0.00]	(0.03) [0.00]	(0.24) [0.00]	(0.06) [0.00]
Observations	13,142	68,731	11,747	45,224
*R*-squared	0.17	0.15	0.22	0.14

*Note:* Coefficients with standard errors in parentheses and exact *p*-values in brackets. Variables unstandardized.

## Discussion

In this research note, I asked whether the major “Western” findings on attitudes to immigration are supported by global evidence. The answer is a qualified yes. The causes of variation in attitudes to immigration have been the subject of extensive scholarship in recent decades, with some factors being repeatedly evidenced and, thus, arguably generalizable. However, these studies had been overwhelmingly from a relatively small selection of “Western” countries. This study made use of recent World Values Survey data across 66 countries globally to test how generalizable findings on the determinants of attitudes to immigration are globally. It found that the most typically identified sociodemographic, economic, and contextual determinants of immigration policy preferences, prejudices, and perceived effects are mostly generalizable globally: age, low income, job worries, rurality, not having an immigrant background, social distrust, national migration rate, feeling unsafe in one’s neighborhood, and national media bias, with gender, education, and left-right self-placement being less so.

Although left-right self-placement is one of the most reliable predictors of immigration attitudes in Western Europe and Anglophone countries, it behaves far more inconsistently outside these contexts. This signals that the ideological spectrum—whether measured on a unidimensional left-right scale or not—does not always provide an appropriate proxy for immigration-related attitudes outside of societies where the political field is structured around economic redistribution or identity-based competition. Consistent with recent work in comparative political behavior, these findings suggest that ideology’s explanatory role may be highly context-dependent, and that other dimensions (such as nationalism, religiosity, or regime support) could substitute for left-right alignment in many parts of the world.

Although education is negatively associated with restrictive immigration attitudes across all regions, the size of this effect is greatest in Western Europe and the Anglosphere. This finding suggests that the now-classic “education cleavage” may not travel as well outside of Western contexts as previously assumed. In many non-Western countries, the effect of education is notably smaller, indicating that tertiary education may have less universal or less politicized implications for immigration-related views. This pattern is consistent with the idea that the “cosmopolitan” values often attributed to higher education in Western societies are rooted not only in educational experience per se, but also in broader political and institutional structures that embed education within specific kinds of cultural or ideological conflicts. These structures—and the discursive centrality of immigration—may be weaker or differently organized in other parts of the world.

Taken together, the analysis shows that some of the most widely cited determinants of immigration attitudes in Western Europe do travel cross-nationally, but not always in the form or magnitude implied by the Western literature. Education, often treated as the defining cleavage in Western debates, is less powerful outside the West; ideological self-placement, another assumed universal, is barely meaningful in most regions once entered alongside other predictors. By contrast, factors emphasized in non-Western research—such as social trust, everyday security, and perceived media credibility (e.g., Gordon and Maharaj 2015; Dennison and Geddes 2021; Joshanloo 2024)—prove to be more consistently patterned across world regions than much of the Western canon assumes. Given the vast and increasingly overdetermined literature on attitudes to immigration, the real task ahead is not to add more predictors, but to test more carefully which ones survive outside their original theoretical habitat. This paper offers one such test and contribution: a deliberately modest assessment of whether the dominant Western determinants replicate elsewhere. The answer is mostly yes.

## Data Availability

Replication data and documentation are available at https://dataverse.harvard.edu/dataset.xhtml?persistentId=doi:10.7910/DVN/VT8NMD.

## Footnotes

1 Andorra, Argentina, Australia, Bangladesh, Armenia, Bolivia, Brazil, Myanmar, Canada, Chile, China, Taiwan (ROC), Colombia, Cyprus, Czechia, Ecuador, Ethiopia, Germany, Greece, Guatemala, Hong Kong (SAR), India, Indonesia, Iran, Iraq, Japan, Kazakhstan, Jordan, Kenya, South Korea, Kyrgyzstan, Lebanon, Libya, Macau (SAR), Malaysia, Maldives, Mexico, Mongolia, Morocco, Netherlands, New Zealand, Nicaragua, Nigeria, Pakistan, Peru, Philippines, Puerto Rico, Romania, Russia, Serbia, Singapore, Slovakia, Vietnam, Zimbabwe, Tajikistan, Thailand, Tunisia, Turkey, Ukraine, Egypt, Great Britain, United States, Uruguay, Uzbekistan, Venezuela, Northern Ireland.

## Appendix A. AAPOR-Required Disclosure Elements

### Data Source

World Values Survey (WVS), Wave 7 (2017–2022)

#### Type of Data and Source

Secondary analysis of survey data. The World Values Survey (WVS) is conducted by the World Values Survey Association using nationally representative samples collected by local fieldwork teams. Data were obtained via the WVS online data archive: https://www.worldvaluessurvey.org.

#### Data Collection Strategy

Face-to-face structured interviews conducted by trained enumerators using a standardized questionnaire translated into national languages. Most interviews were conducted using paper-and-pencil or computer-assisted personal interviewing (CAPI).

#### Research Sponsor and Conductor

The WVS is sponsored by the World Values Survey Association and conducted by a network of affiliated academic institutions and survey agencies in each participating country. Full details of fieldwork organizations are available in country-level technical reports hosted on the WVS website.

#### Measurement Tools/Instruments

The survey instrument consists of a common core questionnaire administered in local languages. The exact wording and ordering of all survey questions analyzed in this manuscript are available at https://www.worldvaluessurvey.org/WVSDocumentationWV7.jsp. Specific question numbers (e.g., Q62, Q71, Q79) are cited in the manuscript and Appendix A.

#### Population Under Study

Adults aged 18 and above residing in private households, with national samples in each of 66 participating countries and societies. Sampling frames vary by country but aim to provide full coverage of the residential adult population.

#### Methods Used to Generate and Recruit the Sample

- All WVS Wave 7 samples are probability-based, using multistage stratified random sampling or stratified cluster sampling, depending on country-specific conditions.
- No quotas were used in respondent selection, except where unavoidable due to field conditions or frame limitations.
- Recruitment relied on interviewer contact at randomly selected households or individuals in public areas, with in-person verification of eligibility.
- Country-level sampling documentation, including sample frames and coverage, are available at https://www.worldvaluessurvey.org under “Technical Fieldwork Reports.”

#### Method and Mode of Data Collection

- Mode: Primarily face-to-face interviews (PAPI or CAPI).
- Languages: Administered in local languages; translation and back-translation validated centrally.
- Duration: Interviews typically lasted 45–60 minutes.

#### Dates of Data Collection

Between 2017 and 2022, with country-specific fieldwork dates available in the WVS Fieldwork Reports.

#### Sample Sizes and Precision

- Approximately 80,000 individuals surveyed in total across 66 countries.
- Country-level sample sizes ranged from ∼1,000 to ∼3,000.
- No single global “margin of error” applies. As the focus of this study is on pooled regression analysis using harmonized data, no sampling error estimates are reported.

#### Whether and How the Data Were Weighted

WVS provides poststratification weights to adjust samples to national population distributions on age, gender, and region. As noted in the main text, analyses in this paper are conducted unweighted for cross-national comparability, with weighted robustness checks available upon request.

#### How the Data Were Processed and Quality Procedures

- WVS centrally validates, cleans, and harmonizes national datasets before release.
- Local fieldwork teams are trained through WVS protocols.
- No additional quality checks (e.g., attention items or recontacts) were imposed post hoc by the authors of this manuscript.
- The variable *H_URBRURAL* (urban/rural distinction) and other demographic harmonizations are included as provided by WVS.

#### Panel Use

Not applicable. WVS is not a panel study.

#### Interviewer Details

Interviews conducted by trained professionals following local WVS procedures. Country-specific interviewer protocols and supervision details are described in national fieldwork reports.

#### Eligibility Screening

Eligibility criteria limited to adults aged 18+; no other screening procedures applied.

#### Study Stimuli

Not applicable; no experimental manipulations or embedded vignettes were used in this analysis.

#### Disposition/Response Rates

WVS does not centrally publish AAPOR-compliant response rates for Wave 7 as a whole. Response rates vary by country and are documented in some country-specific field reports. Therefore, complete disposition data and response rates cannot be provided for all countries in this study and should be regarded as a limitation.

#### Sample Sizes in Analysis

Unweighted sample sizes for each variable appear in table 1 of the manuscript. Variance in *N* reflects item nonresponse and country sample differences.

#### Measurement and Model Specification

All dependent and independent variables used in the modeling are defined in the manuscript text and Appendix A. Statistical code is available upon request.

#### General Statement of Limitations

The WVS uses high-quality, probability-based samples across diverse settings, but cross-country differences in fieldwork practices, local sampling frames, and survey modes may limit direct comparability. The lack of consolidated AAPOR response rate data and incomplete reporting of design-based variance estimation procedures constitute further limitations.

**Table A1.** Four linear regression models predicting restrictive immigration policy preferences with population weights.

	(1)	(2)	(3)	(4)
	Restrictive preferences			
	Sociodemographics and economics		Add attitudes and context	
	W. Europe and English-speaking	Rest of world	W. Europe and English-speaking	Rest of world
Education (ref: none/primary)				
Secondary	−0.08	0.00	−0.03	0.01
	(0.01) [0.00]	(0.02) [0.89]	(0.01) [0.06]	(0.03) [0.64]
Tertiary	−0.33	−0.01	−0.19	0.01
	(0.04) [0.00]	(0.02) [0.81]	(0.02) [0.00]	(0.03) [0.78]
Income decile	−0.01	−0.02	−0.01	−0.03
	(0.00) [0.05]	(0.00) [0.00]	(0.00) [0.04]	(0.01) [0.02]
Job worries	−0.05	−0.04	−0.03	−0.04
	(0.01) [0.00]	(0.02) [0.02]	(0.01) [0.01]	(0.02) [0.03]
Female	−0.05	0.00	−0.04	0.02
	(0.01) [0.00]	(0.01) [0.95]	(0.02) [0.07]	(0.02) [0.29]
Immigrant	−0.01	−0.15	−0.06	−0.10
	(0.02) [0.51]	(0.06) [0.02]	(0.01) [0.00]	(0.07) [0.18]
Immigrant parent	−0.16	−0.01	−0.15	−0.05
	(0.04) [0.00]	(0.05) [0.82]	(0.04) [0.01]	(0.03) [0.10]
Age	0.01	0.00	0.00	0.00
	(0.00) [0.00]	(0.00) [0.00]	(0.00) [0.00]	(0.00) [0.00]
Social distrust			0.24	0.24
			(0.02) [0.00]	(0.04) [0.00]
Feel close to country			−0.04	0.02
			(0.01) [0.00]	(0.03) [0.57]
Left-right self-placement			0.09	0.01
			(0.01) [0.00]	(0.01) [0.06]
National migration rate			0.01	0.02
			(0.00) [0.17]	(0.00) [0.00]
Media unbias			−0.16	−0.22
			(0.00) [0.00]	(0.01) [0.00]
Feel safe in neighborhood			−0.06	0.03
			(0.02) [0.03]	(0.01) [0.02]
Rurality			0.09	0.04
			(0.04) [0.05]	(0.02) [0.05]
Country dummy variables	YES	YES	YES	YES
SE clustered by country	YES	YES	YES	YES
Individual weight	YES	YES	YES	YES
Population weight	YES	YES	YES	YES
Constant	2.43	2.42	1.92	2.08
	(0.02) [0.00]	(0.04) [0.00]	(0.08) [0.00]	(0.10) [0.00]
Observations	13,030	68,867	11,662	45,177
*R*-squared	0.08	0.06	0.20	0.07

*Note:* Coefficients with standard errors in parentheses and exact *p*-values in brackets. Variables unstandardized.

**Table A2.** Linear regression models predicting restrictive immigration policy preferences by world region.

	(1)	(2)	(3)	(4)	(5)	(6)	(7)	(8)	(9)
	Restrictive preferences								
	Western Europe	CEEC and Balkan	Former Soviet Union	English-speaking	Latin America	MENA	Sub-Saharan Africa	South and Southeast Asia	East Asia
Education (ref: none/primary)									
Secondary	−0.07	−0.10	0.05	−0.04	−0.07	−0.01	0.08	0.02	−0.00
	(0.04) [0.20]	(0.04) [0.08]	(0.03) [0.18]	(0.03) [0.17]	(0.04) [0.07]	(0.01) [0.65]	(0.04) [0.13]	(0.04) [0.69]	(0.03) [0.93]
Tertiary	−0.24	−0.19	0.04	−0.22	−0.19	−0.02	0.04	0.05	−0.05
	(0.07) [0.02]	(0.04) [0.02]	(0.05) [0.45]	(0.05) [0.00]	(0.06) [0.01]	(0.03) [0.59]	(0.10) [0.73]	(0.04) [0.28]	(0.04) [0.17]
Income decile	−0.00	−0.03	−0.02	−0.01	−0.01	−0.02	−0.01	−0.01	−0.02
	(0.01) [0.78]	(0.01) [0.05]	(0.01) [0.04]	(0.01) [0.29]	(0.00) [0.09]	(0.01) [0.03]	(0.01) [0.25]	(0.01) [0.03]	(0.01) [0.09]
Job worries	−0.07	−0.07	−0.06	−0.05	−0.01	−0.02	0.03	−0.03	−0.05
	(0.02) [0.05]	(0.02) [0.06]	(0.01) [0.00]	(0.01) [0.00]	(0.01) [0.53]	(0.01) [0.15]	(0.04) [0.44]	(0.02) [0.13]	(0.01) [0.00]
Female	−0.05	−0.00	0.04	−0.03	0.03	0.01	0.03	0.02	0.02
	(0.02) [0.10]	(0.03) [0.88]	(0.03) [0.24]	(0.02) [0.14]	(0.02) [0.21]	(0.01) [0.27]	(0.03) [0.42]	(0.03) [0.55]	(0.02) [0.26]
Immigrant	−0.12	−0.11	0.00	−0.04	−0.24	0.05	−0.20	−0.36	−0.13
	(0.03) [0.02]	(0.14) [0.48]	(0.04) [0.94]	(0.02) [0.14]	(0.13) [0.10]	(0.05) [0.37]	(0.09) [0.09]	(0.15) [0.04]	(0.02) [0.00]
Immigrant parent	−0.10	0.00	−0.13	−0.12	−0.02	−0.16	−0.11	0.05	−0.03
	(0.04) [0.07]	(0.13) [0.98]	(0.07) [0.13]	(0.03) [0.01]	(0.07) [0.79]	(0.06) [0.02]	(0.04) [0.06]	(0.13) [0.68]	(0.05) [0.57]
Age	0.00	0.01	0.01	0.01	0.00	0.00	0.00	0.01	0.00
	(0.00) [0.03]	(0.00) [0.00]	(0.00) [0.00]	(0.00) [0.00]	(0.00) [0.00]	(0.00) [0.03]	(0.00) [0.30]	(0.00) [0.00]	(0.00) [0.09]
Country dummy variables	YES	YES	YES	YES	YES	YES	YES	YES	YES
SE clustered by country	YES	YES	YES	YES	YES	YES	YES	YES	
Individual weight	YES	YES	YES	YES	YES	YES	YES	YES	NO
Population weight	YES	YES	YES	YES	YES	YES	YES	YES	YES
Constant	2.54	2.72	2.11	2.47	2.37	2.77	2.62	2.21	2.87
	(0.12) [0.00]	(0.08) [0.00]	(0.07) [0.00]	(0.03) [0.00]	(0.06) [0.00]	(0.05) [0.00]	(0.10) [0.00]	(0.09) [0.00]	(0.09) [0.00]
Observations	4,374	4,084	8,295	10,200	16,342	10,805	5,965	13,663	9,725
*R*-squared	0.16	0.07	0.12	0.07	0.10	0.07	0.03	0.06	0.08

*Note:* Coefficients with standard errors in parentheses and exact *p*-values in brackets. Variables unstandardized.

**Table A3.** Linear regression models predicting restrictive immigration policy preferences by world region with attitudinal and contextual predictors.

	(1)	(2)	(3)	(4)	(5)	(6)	(7)	(8)	(9)
	Restrictive preferences								
	Western Europe	CEEC and Balkan	Former Soviet Union	English-speaking	Latin America	MENA	Sub-Saharan Africa	South and Southeast Asia	East Asia
Education (ref: none/primary)									
Secondary	−0.05	−0.09	0.04	−0.02	−0.07	0.00	0.09	0.06	0.02
	(0.05) [0.38]	(0.04) [0.14]	(0.01) [0.07]	(0.03) [0.53]	(0.04) [0.07]	(0.03) [0.94]	(0.04) [0.13]	(0.02) [0.03]	(0.02) [0.42]
Tertiary	−0.19	−0.16	0.04	−0.14	−0.18	−0.00	0.16	0.06	−0.01
	(0.04) [0.02]	(0.05) [0.06]	(0.02) [0.20]	(0.03) [0.01]	(0.06) [0.01]	(0.04) [0.98]	(0.08) [0.13]	(0.04) [0.15]	(0.03) [0.66]
Income decile	−0.00	−0.03	−0.02	−0.01	−0.01	−0.02	−0.01	−0.02	−0.02
	(0.00) [0.49]	(0.00) [0.01]	(0.01) [0.14]	(0.01) [0.44]	(0.01) [0.39]	(0.01) [0.31]	(0.01) [0.52]	(0.01) [0.02]	(0.01) [0.09]
Job worries	−0.08	−0.06	−0.06	−0.03	−0.02	−0.02	0.03	−0.03	−0.04
	(0.02) [0.03]	(0.01) [0.01]	(0.02) [0.09]	(0.01) [0.01]	(0.01) [0.09]	(0.02) [0.39]	(0.02) [0.33]	(0.02) [0.16]	(0.01) [0.05]
Female	−0.05	−0.01	0.04	−0.01	0.02	0.01	0.04	0.03	0.02
	(0.02) [0.10]	(0.03) [0.80]	(0.05) [0.44]	(0.02) [0.63]	(0.02) [0.26]	(0.02) [0.55]	(0.04) [0.37]	(0.03) [0.26]	(0.02) [0.53]
Immigrant	−0.13	−0.09	0.05	−0.06	−0.29	0.13	−0.10	−0.36	−0.14
	(0.05) [0.08]	(0.15) [0.58]	(0.06) [0.47]	(0.01) [0.01]	(0.15) [0.08]	(0.17) [0.50]	(0.33) [0.78]	(0.20) [0.11]	(0.03) [0.01]
Immigrant parent	−0.08	−0.01	−0.00	−0.11	0.01	−0.27	−0.14	−0.09	−0.05
	(0.04) [0.16]	(0.14) [0.97]	(0.05) [0.96]	(0.03) [0.03]	(0.08) [0.95]	(0.08) [0.05]	(0.05) [0.08]	(0.04) [0.09]	(0.06) [0.48]
Age	0.00	0.01	0.00	0.01	0.00	0.00	0.00	0.01	0.00
	(0.00) [0.08]	(0.00) [0.00]	(0.00) [0.00]	(0.00) [0.01]	(0.00) [0.01]	(0.00) [0.04]	(0.00) [0.64]	(0.00) [0.00]	(0.00) [0.21]
Social distrust	0.23	0.16	0.22	0.21	0.11	0.13	0.15	0.27	0.13
	(0.02) [0.00]	(0.07) [0.11]	(0.06) [0.04]	(0.02) [0.00]	(0.07) [0.12]	(0.05) [0.07]	(0.10) [0.25]	(0.05) [0.00]	(0.05) [0.07]
Feel close to country	−0.03	−0.05	0.01	−0.01	−0.01	−0.02	−0.02	0.01	−0.03
	(0.02) [0.26]	(0.02) [0.13]	(0.03) [0.88]	(0.03) [0.83]	(0.01) [0.33]	(0.05) [0.72]	(0.03) [0.56]	(0.03) [0.61]	(0.02) [0.31]
Left-right self-placement	0.06	−0.00	−0.01	0.07	0.01	−0.02	0.01	−0.00	−0.01
	(0.03) [0.10]	(0.01) [0.98]	(0.01) [0.34]	(0.02) [0.01]	(0.01) [0.02]	(0.01) [0.20]	(0.01) [0.57]	(0.01) [0.82]	(0.01) [0.56]
National migration rate	0.03	−0.03	0.20	0.01	0.01	−0.08	−0.00	0.17	0.00
	(0.02) [0.14]	(0.01) [0.03]	(0.01) [0.00]	(0.00) [0.05]	(0.00) [0.00]	(0.01) [0.01]	(0.01) [0.84]	(0.01) [0.00]	(0.02) [0.91]
Media unbias	−0.21	0.13	−0.18	−0.17	−0.19	−0.35	−0.04	1.36	−0.21
	(0.06) [0.04]	(0.01) [0.00]	(0.01) [0.00]	(0.01) [0.00]	(0.01) [0.00]	(0.00) [0.00]	(0.02) [0.08]	(0.07) [0.00]	(0.08) [0.05]
Feel safe in neighborhood	−0.02	0.05	0.00	−0.04	0.00	0.06	−0.00	0.04	0.12
	(0.01) [0.26]	(0.02) [0.12]	(0.02) [0.96]	(0.02) [0.10]	(0.01) [0.58]	(0.03) [0.15]	(0.01) [0.95]	(0.03) [0.28]	(0.04) [0.02]
Rurality	0.02	0.09	0.05	0.03	0.04	0.11	0.07	−0.04	0.12
	(0.05) [0.70]	(0.06) [0.24]	(0.04) [0.35]	(0.03) [0.40]	(0.02) [0.12]	(0.06) [0.17]	(0.03) [0.09]	(0.02) [0.11]	(0.03) [0.01]
Country dummy variables	YES	YES	YES	YES	YES	YES	YES	YES	YES
SE clustered by country	YES	YES	YES	YES	YES	YES	YES	YES	
Individual weight	YES	YES	YES	YES	YES	YES	YES	YES	NO
Population weight	YES	YES	YES	YES	YES	YES	YES	YES	YES
Constant	2.96	2.28	2.42	2.13	2.37	2.76	2.09	1.04	2.45
	(0.23) [0.00]	(0.11) [0.00]	(0.20) [0.00]	(0.16) [0.00]	(0.07) [0.00]	(0.17) [0.00]	(0.10) [0.00]	(0.13) [0.00]	(0.22) [0.00]
Observations	3,242	3,486	3,476	9,554	13,127	4,906	3,877	9,307	6,998
*R*-squared	0.22	0.08	0.11	0.14	0.10	0.12	0.02	0.10	0.09

*Note:* Coefficients with standard errors in parentheses and exact *p*-values in brackets. Variables unstandardized.

**Table A4.** Four linear regression models predicting anti-immigrant prejudice with population weights.

	(1)	(2)	(3)	(4)
	Anti-immigrant prejudice			
	Sociodemographics and economics		Add attitudes and context	
	W. Europe and English-speaking	Rest of world	W. Europe and English-speaking	Rest of world
Education (ref: none/primary)				
Secondary	0.01	−0.01	0.01	−0.01
	(0.02) [0.79]	(0.01) [0.18]	(0.02) [0.56]	(0.02) [0.65]
Tertiary	−0.04	−0.06	−0.02	−0.07
	(0.02) [0.04]	(0.02) [0.00]	(0.02) [0.40]	(0.02) [0.79]
Income decile	0.00	−0.00	0.00	0.00
	(0.00) [0.05]	(0.00) [0.13]	(0.00) [0.03]	(0.00) [0.19]
Job worries	−0.02	0.00	−0.01	0.01
	(0.00) [0.00]	(0.00) [0.58]	(0.00) [0.02]	(0.01) [0.09]
Female	−0.02	0.00	−0.02	0.01
	(0.00) [0.00]	(0.00) [0.47]	(0.00) [0.00]	(0.01) [0.14]
Immigrant	−0.03	−0.04	−0.04	−0.04
	(0.00) [0.00]	(0.03) [0.18]	(0.01) [0.00]	(0.03) [0.70]
Immigrant parent	−0.04	−0.04	−0.04	−0.01
	(0.01) [0.00]	(0.03) [0.14]	(0.01) [0.00]	(0.02) [0.03]
Age	0.00	0.00	0.00	0.00
	(0.00) [0.01]	(0.00) [0.06]	(0.00) [0.06]	(0.00) [0.10]
Social distrust			0.06	−0.05
			(0.01) [0.00]	(0.03) [0.87]
Feel close to country			−0.00	−0.00
			(0.01) [1.00]	(0.00) [0.83]
Left-right self-placement			0.02	0.00
			(0.00) [0.00]	(0.00) [0.00]
National migration rate			0.00	−0.00
			(0.00) [0.01]	(0.00) [0.25]
Media unbias			−0.03	0.01
			(0.00) [0.00]	(0.01) [0.04]
Feel safe in neighborhood			−0.02	−0.03
			(0.00) [0.00]	(0.02) [0.72]
Rurality			−0.00	−0.00
			(0.01) [0.40]	(0.01) [0.02]
Country dummy variables	YES	YES	YES	YES
SE clustered by country	YES	YES	YES	YES
Individual weight	YES	YES	YES	YES
Population weight	YES	YES	YES	YES
Constant	0.10	0.05	0.00	0.18
	(0.02) [0.00]	(0.01) [0.00]	(0.03) [0.89]	(0.07)
Observations	13,092	69,492	11,657	45,192
*R*-squared	0.03	0.13	0.06	0.14

*Note:* Coefficients with standard errors in parentheses and exact *p*-values in brackets. Variables unstandardized.

**Table A5.** Four linear regression models predicting belief that immigration has been good for the country’s development with population weights.

	(1)	(2)	(3)	(4)
	Immigration has been good for country’s development			

	Sociodemographics and economics		Add psychology and context	
	W. Europe and English-speaking	Rest of world	W. Europe and English-speaking	Rest of world
Education (ref: none/primary)				
Secondary	−0.01	−0.05	−0.08	−0.06
	(0.05) [0.85]	(0.02) [0.01]	(0.06) [0.22]	(0.02) [0.00]
Tertiary	0.32	−0.01	0.15	−0.04
	(0.05) [0.00]	(0.02) [0.53]	(0.07) [0.07]	(0.02) [0.10]
Income decile	0.03	0.05	0.04	0.05
	(0.01) [0.00]	(0.02) [0.01]	(0.01) [0.00]	(0.02) [0.01]
Job worries	0.06	0.01	0.04	−0.00
	(0.01) [0.00]	(0.01) [0.38]	(0.01) [0.00]	(0.01) [0.85]
Female	−0.07	0.02	−0.09	−0.00
	(0.04) [0.13]	(0.02) [0.26]	(0.04) [0.05]	(0.01) [1.00]
Immigrant	0.24	0.32	0.31	0.29
	(0.04) [0.00]	(0.08) [0.00]	(0.05) [0.00]	(0.11) [0.01]
Immigrant parent	0.26	0.07	0.24	0.16
	(0.08) [0.01]	(0.07) [0.35]	(0.08) [0.02]	(0.05) [0.00]
Age	−0.00	−0.00	−0.00	−0.00
	(0.00) [0.00]	(0.00) [0.00]	(0.00) [0.49]	(0.00) [0.01]
Social distrust			−0.27	−0.16
			(0.05) [0.00]	(0.03) [0.00]
Feel close to country			−0.03	0.03
			(0.01) [0.05]	(0.03) [0.28]
Left-right self-placement			−0.11	−0.01
			(0.01) [0.00]	(0.01) [0.44]
National migration rate			−0.15	−0.02
			(0.00) [0.00]	(0.00) [0.00]
Media unbias			0.41	0.19
			(0.01) [0.00]	(0.02) [0.00]
Feel safe in neighborhood			0.02	−0.06
			(0.02) [0.33]	(0.05) [0.28]
Rurality			−0.09	0.02
			(0.05) [0.14]	(0.03) [0.52]
Country dummy variables	YES	YES	YES	YES
SE clustered by country	YES	YES	YES	YES
Individual weight	YES	YES	YES	YES
Population weight	YES	YES	YES	YES
Constant	3.28	2.96	4.14	3.15
	(0.13) [0.00]	(0.05) [0.00]	(0.11) [0.00]	(0.09) [0.00]
Observations	13,142	68,731	11,747	45,224
*R*-squared	0.14	0.10	0.22	0.12

*Note:* Coefficients with standard errors in parentheses and exact *p*-values in brackets. Variables unstandardized.
